# 2D Ionic Liquid‐Like State of Charged Rare‐Earth Clusters on a Metal Surface

**DOI:** 10.1002/advs.202308813

**Published:** 2024-01-24

**Authors:** Daniel Trainer, Alex Taekyung Lee, Sanjoy Sarkar, Vijay Singh, Xinyue Cheng, Naveen K. Dandu, Kyaw Zin Latt, Shaoze Wang, Tolulope Michael Ajayi, Sineth Premarathna, David Facemyer, Larry A. Curtiss, Sergio E. Ulloa, Anh T. Ngo, Eric Masson, Saw Wai Hla

**Affiliations:** ^1^ Nanoscience and Technology Division Argonne National laboratory Lemont IL 60439 USA; ^2^ Chemical Engineering Department University of Illinois at Chicago Chicago IL 60608 USA; ^3^ Materials Science Division Argonne National laboratory Lemont IL 60439 USA; ^4^ Nanoscale and Quantum Phenomena Institute and Department of Physics and Astronomy Ohio University Athens OH 45701 USA; ^5^ Department of Chemistry and Biochemistry Ohio University Athens OH 45701 USA; ^6^ Present address: Department of Physics GITAM School of Science Bengaluru Karnataka 561203 India

**Keywords:** Au(111) surface, ionic liquid, rare‐earth metals, scanning tunneling microscopy, triflate anions

## Abstract

Rare‐earth complexes are vital for separation chemistry and useful in many advanced applications including emission and energy upconversion. Here, 2D rare‐earth clusters having net charges are formed on a metal surface, enabling investigations of their structural and electronic properties on a one‐cluster‐at‐a‐time basis using scanning tunneling microscopy. While these ionic complexes are highly mobile on the surface at ≈100 K, their mobility is greatly reduced at 5 K and reveals stable and self‐limiting clusters. In each cluster, a pair of charged rare‐earth complexes formed by electrostatic and dispersive interactions act as a basic unit, and the clusters are chiral. Unlike other non‐ionic molecular clusters formed on the surfaces, these rare‐earth clusters show mechanical stability. Moreover, their high mobility on the surface suggests that they are in a 2D liquid‐like state.

## Graphical abstract

2D ionic rare‐earth clusters are formed on Au(111) surface where a pair of charged rare‐earth complexes act as a basic unit, and the clusters are chiral. Their high mobility on the surface suggests they are in a 2D liquid‐like state, which has implications for material designs for potential applications including separation, emission, and quantum sciences.

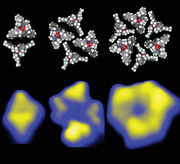

## 1 Introduction

Rare‐earth elements are vital for many advanced technological applications including emission devices,^[^ ^1^, ^2^ ^]^ powerful magnets,^[^ ^3^ ^]^ and quantum computations^[^ ^4^, ^5^ ^]^ owing to the unique properties of their 4f electron configurations. Despite being known as “rare‐earth,” they are abundant in the earth's crust, but their separation process is very expensive.^[^ ^6^ ^]^ The economically and environmentally viable separation of rare earth is considered to be one of the most important processes in the industry.^[^ ^7^ ^]^ For separation purposes, rare‐earth ions coordinated to organic ligands are important,^[^ ^8^, ^9^ ^]^ especially the ones containing counterions are of particular interest.^[^ ^10^, ^11^ ^]^ Such rare‐earth coordination complexes are also uniquely advantageous in developing rational blueprints for the design and synthesis of new structures with improved functions for potential applications.^[^ ^12^, ^13^ ^]^ However, the role of counterions and the formation of rare‐earth complex clusters have yet to be explored at the single complex level. Recently, it has been demonstrated that by efficient protection of the surrounding ligands, the rare‐earth ions in the complexes together with counterions can remain charged even on a metallic surface.^[^ ^14^ ^]^ Generally, charged molecular complexes are difficult to form on metal surfaces and single‐molecule studies are usually performed on insulating substrates.^[^ ^15^, ^16^ ^]^ Therefore, maintaining net charges in rare‐earth complexes on metallic surfaces^[^ ^14^, ^17^ ^]^ opens opportunities to investigate their properties for potential applications. Here, using a ligand‐bound rare‐earth triflate salt, we create charged rare‐earth clusters with even numbers of units on a Au(111) surface at low temperatures. The formation of self‐limiting clusters in a controlled and isolated environment enables investigations of their structural and electronic properties on a one‐cluster‐at‐a‐time basis using scanning tunneling microscopy (STM) and tunneling spectroscopy methods. The experimental findings are supported by density functional theory (DFT) calculations, revealing their energetics and stability.

## 2 Results

**Figure 1:**
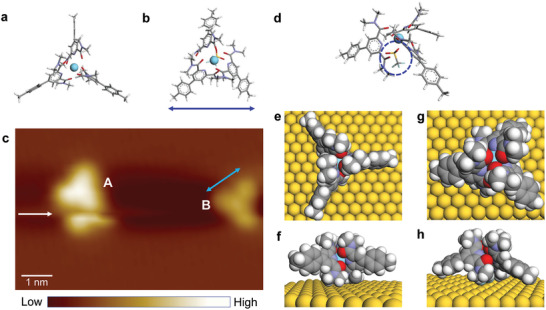
Structures of lanthanum complexes. a) A model of [La(pcam)_3_]^3+^. b) A model of complex [La(pcam)_3_X]^2+^. c) STM image of [La(pcam)_3_]^3+^ (A) and [La(pcam)_3_X]^2+^ (B) on a Cu(111) surface. The blue arrow indicates a location where the [La(pcam)_3_]^3+^ complex is moved during scanning while the white arrow indicates the shorter side of the [La(pcam)_3_X]^2+^ complex. (Tunneling parameters: *V*_t_ = 1 V, *I*_t_ = 2.6 × 10^−11^ A). d) An angled view of [La(pcam)_3_X]^2+^ where the triflate counterion is indicated with a dashed oval. e) DFT calculated adsorption geometry of [La(pcam)_3_]^3+^ on Au(111) surface in top view, and f) in side view. g) DFT calculated adsorption geometry of [La(pcam)_3_X]^2+^ on Au(111) surface in top view, and h) in a side view.

The coordination complex used here is [La(pcam)_3_]^3+^—lanthanum(III) tris(2,6‐pyridine di carboxamide) (**Figure** **1**).^[^ ^18^ ^]^ La(pcam)_3_ has three equivalent ligand arms in a planar and a distorted D_3h_ geometry (Figure 1a). The experiments are performed on two separate metallic surfaces, Cu(111) and Au(111). The selection of the surface is based on the reactivity of the substrate. The complexes weakly bind to both surfaces, however, stronger binding to Cu(111) than to Au(111). Thus Cu(111) is selected to acquire stable individual complex images while Au(111) is used to promote a higher mobility of the complexes to form ionic clusters.

To investigate the structures of complexes using STM imaging, La(pcam)_3_ triflate (CF_3_SO_3_^−^) salt was initially deposited onto an atomically clean Cu(111) surface. STM image (Figure 1c) shows two isolated complexes, which are labeled as “A” and “B.” The arrow in A indicates the movement of the molecule during scanning in which a part of the molecule is again reimaged. Based on the shape of the structure, “A” is attributed to a pristine La(pcam)_3_, which we will describe as [La(pcam)_3_]^3+^ complex. In a previous study of similar complexes formed with europium (Eu) ion, Eu(pcam)_3_, we have shown that the counterion (CF_3_SO_3_^−^) preferentially binds to the molecule underneath, and the resulting complex, [Eu(pcam)_3_X]^2+^ where X = CF_3_SO_3_^−^ has a distorted triangular shape with one of its side lengths shorter,^[^ ^14^ ^]^ which is similar to the structure of “B” here (Figure 1b,c). Thus, “B” is assigned as a [La(pcam)_3_X]^2+^ complex. Moreover, the [La(pcam)_3_X]^2+^ complex appears slightly darker than [La(pcam)_3_]^3+^. Such a darker contrast can be associated with incorporating a negative charge.^[^ ^14^, ^15^, ^16^ ^]^ Since the [La(pcam)_3_X]^2+^ complex has a negative counterion underneath, the intensity of tunneling current is reduced as compared to [La(pcam)_3_]^3+^ due to screening. As a result, the complex appears darker. Furthermore, STM images reveal higher mobility of the [La(pcam)_3_]^3+^ complexes even on the Cu(111) surface at 5K substrate temperature (Figure 1c). The DFT calculated geometries of [La(pcam)_3_]^3+^ and [La(pcam)_3_X]^2+^ complexes are presented in Figure 1e–h.

**Figure 2:**
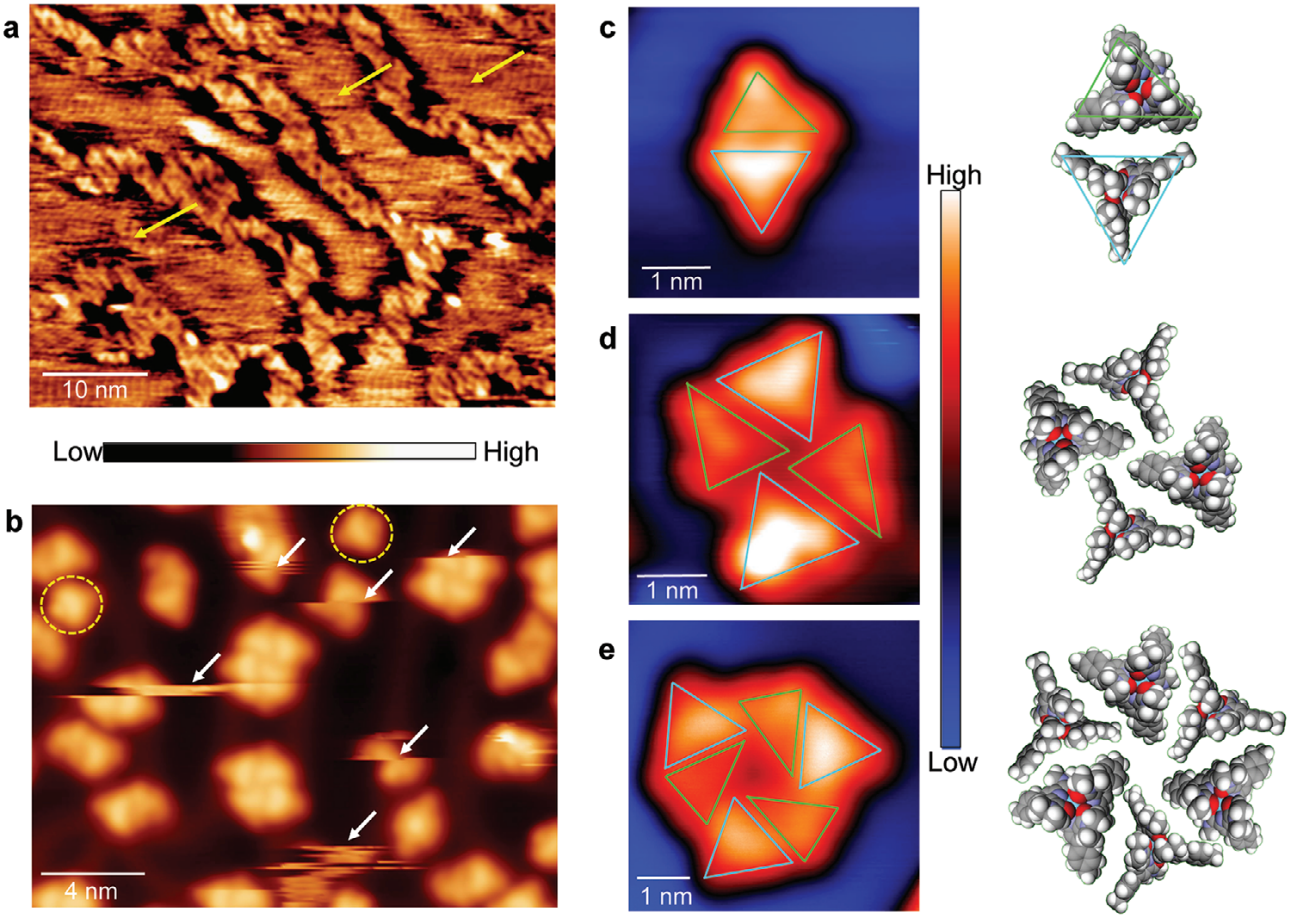
Rare‐earth clusters. a) An STM image acquired at 120 K substrate temperature on Au(111) shows the ionic liquid‐like condition of deposited salt. The arrows indicate highly fluid ionic species. (Tunneling parameters: *V*_t_ = 1.8 V, *I*_t_ = 6 × 10^−11^ A). b) An STM image acquired at 5 K substrate temperature on Au(111) shows the clusters of rare‐earth complexes. The arrows indicate the mobility of the clusters while the ovals mark the complexes having two triflate ions. (Tunneling parameters: *V*_t_ = 1 V, *I*_t_ = 3.8 × 10^−11^ A). c–e) STM images of two, four, and six‐unit clusters and corresponding models, respectively. [La(pcam)_3_]^3+^ and [La(pcam)_3_X]^2+^ complexes are marked with light blue and light green triangles, respectively. (Tunneling parameters: *V*_t_ = 0.5 V, *I*_t_ = 3.3 × 10^−11^ A for (c); *V*_t_ = −2 V, *I*_t_ = 3.8 × 10^−11^ A for (d); and *V*_t_ = 1 V, *I*_t_ = 1.2 × 10^−11^ A for (e)).

When STM images are acquired on the sample having ≈0.7 monolayer coverage of La(pcam)_3_ salt on the Au(111) surface at ≈120 K substrate temperature, the complexes are highly mobile on the surface creating noisy image patterns (**Figure** **2**a). This behavior can be attributed to the quasi‐liquid state formed by the mobile La ionic salt complexes, similar to melting,^[^ ^19^ ^]^ on the Au(111) surface. Due to low vapor pressure, ionic liquids formed by cationic and anionic species with melting points typically below 373 K are non‐volatile. Thus they are well suited for environmentally friendly synthesis and separation.^[^ ^20^ ^]^ Here, the neutralizing anionic component can screen charges, giving stability to the 2D liquid‐like state. The net charges of the [La(pcam)_3_]^3+^ and [La(pcam)_3_X]^2+^ complexes result in a long‐range electrostatic attraction to the metallic surface. The screening by the mobile electrons in the metal produces an attractive potential toward the surface, which in the classical limit can be seen as arising from an image charge akin to ionic bonding between the complex and surface.

When the substrate temperature is lowered to 5 K, the ionic liquid‐like state is frozen, and STM images reveal small 2D crystallites in the form of molecular clusters scattered on the surface (Figure 2b). Note that isolated complexes could not be imaged on the Au(111) surface even at ≈5 K substrate temperature because of their high mobility. Although individual clusters can now be imaged with molecular resolution, they are still relatively mobile on the surface (Figure 2b and Supporting Information). In addition, a few complexes with two triflate counterions, [La(pcam)_3_X_2_]^+^, where one is located underneath while the other is attached to a side of the molecule, are also found on the surface. Such complexes with two counterions can be formed by europium (Eu^III^) complexes on Au(111) surface as well.^[^ ^14^ ^]^

Most of the observed molecular clusters are composed of even numbers of units 2, 4, and 6 (Figure 2c–e). Odd unit aggregates having 3 or 5 complexes can occasionally be found on the surface as well. However, they appear to be formed by an additional complex getting stuck to a two‐unit or a four‐unit cluster, and they are not mobile like the even unit clusters (Supporting Information). On the surface, two‐ and four‐unit clusters are abundant; however, very few six‐unit clusters are found. Here, the basic unit of the molecular clusters is a pair formed by a [La(pcam)_3_]^3+^ complex, and a [La(pcam)_3_X]^2+^ complex, which can be clearly distinguished in STM images (Figure 2c); [La(pcam)_3_]^3+^ is more symmetric and appears brighter while the [La(pcam)_3_X]^2+^ complex is less bright overall and has a distorted trigonal shape (Figure 2c), similar to that observed on Cu(111) (Figure 1h). By laterally stacking two pairs of basic units together, a four‐unit cluster is formed (Figure 2d) while a six‐unit cluster is composed of three basic unit pairs rotated by 120° to each other (Figure 2e). From these clusters, it is evident that the units are paired in an alternating arrangement between [La(pcam)_3_]^3+^ and [La(pcam)_3_X]^2+^ complexes.

DFT calculations reveal that the La ion coordinated at the center of [La(pcam)_3_]^3+^ and [La(pcam)_3_X]^2+^ complexes are located relatively far distances, 7.9 and 8.9 Å respectively, from the top Au surface atom (Supporting Information). Moreover, the 4f orbitals of La ion are empty. They are not involved in chemical bonding with the ligands, prohibiting an efficient charge transfer between the La ion and the substrate. Calculations unveil only a negligible amount of charge transfer between the surface and the complexes. Thus, their net charges on Au(111) remain almost the same as in the gas phase, as seen in the Eu‐based complexes.^[^ ^14^ ^]^ Bader charge analysis of the [La(pcam)_3_]^3+^ and [La(pcam)_3_X]^2+^ complexes for the gas phase as well as on Au(111) surface are provided in **Table** **1**. In the gas phase, the La ion of the La(pcam)_3_ complex has a +3e oxidation state.

**Table 1:** The valence charges of La(pacam)_3_ and triflate counterion.

	Gas Phase	On Au(111)
La(pcam)_3_	+3.00e	+2.73e
[La(pcam)_3_X]^2+^	+2.87e	+2.84e
Triflate ion (X) of [La(pcam)_3_X]^2+^	−0.87e	−0.90e

After adsorption of the molecule on Au(111), the substrate transfers −0.27e to the complex, and therefore the La ion attains a net charge of +2.73e. For the [La(pcam)_3_X]^2+^ complex in the gas phase, the La and triflate ions have net charges of +2.87e and −0.87e, respectively. After adsorption on Au(111), the net charge of the La ion in the complex slightly decreases to +2.84e while the negative charge of the triflate counterion slightly increases to −0.90e (Supporting Information S4). Thus, the net charge of the entire complex is +1.94e on Au(111). This means that individual cation and anion units in the complex maintain their own charges. Therefore, these ionic clusters are different from a class of materials known as donor–acceptor charge transfer complexes^[^ ^21^, ^22^, ^23^ ^]^ where charge transfer from the donor to the acceptor molecules stabilizes the materials.

**Figure 3:**
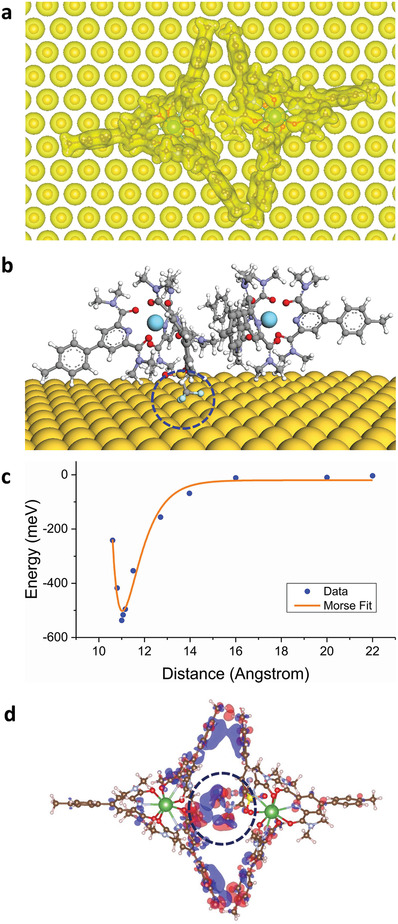
Calculated structure of a complex pair on Au(111). a) A DFT calculated charge density plot of a complex pair adsorbed on Au(111) slab showing optimal geometry. b) A side view of the complex pair where the two La atoms (light blue balls) are separated by ≈11 Å. The position of the triflate counterion is shown by a dashed oval. c) Calculated potential energy as a function of distance between the two La atoms of complex pair. d) Calculated charge density plot showing local electrostatic interactions (indicated with an oval) between the two complexes. Blue: charge gain, and red: charge lost.

In order to confirm the nature of these structures and interactions, DFT calculations are performed on a pair of geometrically relaxed [La(pcam)_3_]^3+^ and [La(pcam)_3_X]^2+^ complexes in the gas phase as well as on the Au(111) surface (**Figure** **3**, and Supporting Information S4). The calculated optimum geometry configuration of the complex pair (Figure 3a) matches with the structure observed in STM images (Figure 2c). In this pair formation, the negatively charged counterion of [La(pcam)_3_X]^2+^ is located slightly toward the [La(pcam)_3_]^3+^ complex (Figure 3b). To check the binding energy between the complexes, total energy calculations are performed by physically displacing the two complexes at various distances. The resultant total energy plot as a function of the distance between the two La ions in the complexes reveals a deep potential well characteristic (Figure 3c). From a Morse potential fit using the relation

(1) $$U\left(r\right)=E_{b}{\left[1-e^{-\alpha \left(r-r_{e}\right)}\right]}^{2}$$

the binding energy between the two complexes is determined to be *E*_b_ ≈ 500 meV. Here *r* is the distance between the two La ions in the complexes, and the equilibrium distance between the two La ions is found as *r_e_* ≈ 11Å. The width of the potential, *α*, is 1.3 Å^−1^. We note that it is essential to include dispersive forces in calculating the inter‐cluster potential because the short‐range interactions between the tolyl substituents in each complex, as shown in Figure 3c, are important for the cluster pairings. In addition, the key for cluster pairing is the local electrostatic interactions between the two complexes. This interaction is originated by the gain and the loss of the net charges at the end methyl groups at the center of the interface (Figure 3d). In order to achieve such a locking mechanism, the complexes must be positioned at a close distance. The La^3+^ ion is strongly screened by the surrounding ligands. In addition, the existence of the image charge on Au(111) surface further adds to the screening. The high mobility of the complexes on Au(111), together with the screening of the positive ion, enables the two complexes to reach a close enough distance to lock in the local electrostatic and dispersive interactions to form such clusters. The net charge gain and charge lost pairing here occur due to the geometry and the different degrees of screening between [La(pcam)_3_X]^2+^ and [La(pcam)_3_]^3+^ complexes. Such a scenario cannot be present between two similar complexes, such as two [La(pcam)_3_X]^2+^ or [La(pcam)_3_]^3+^ complexes where the like charges at the interface will lead to repulsion. Thus, we do not observe clusters formed between two complexes having the same charges.

**Figure 4:**
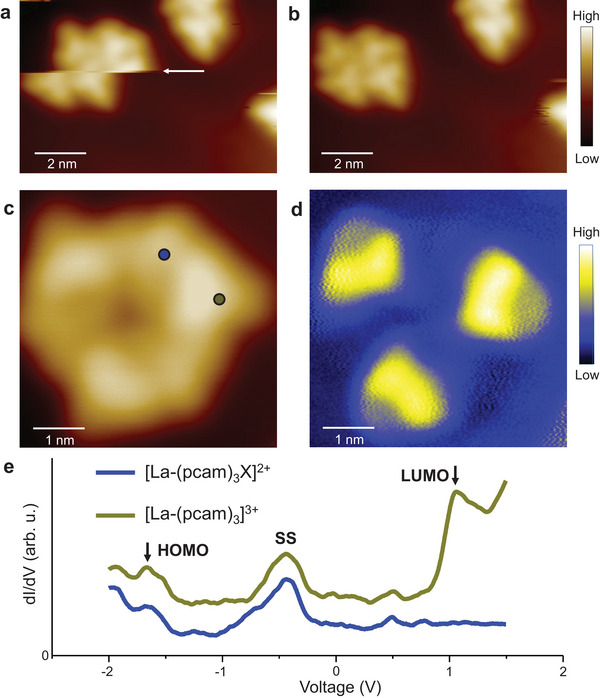
Cluster mobility and electronic structures. a) An STM image shows an abrupt lateral movement (indicated with an arrow) of a four‐unit cluster during scanning. b) When the same area is imaged again, the cluster is laterally displaced to the left but remains intact. (Tunneling parameters for “a” and “b”: *V*_t_ = 1.0 V, *I*_t_ = 3.8 × 10^−11^ A). c) STM image of a six‐unit cluster (Tunneling parameters: *V*_t_ = 1.0 V, *I*_t_ = 1.2 × 10^−11^ A) and d) a simultaneously acquired d*I/*d*V* map at +1 V reveals bright protrusions (yellow regions) on [La(pcam)_3_]^3+^ complexes in the cluster while the signal is absent on the [La(pcam)_3_X]^2+^ complexes. e) d*I/*d*V* tunneling spectroscopy of [La(pcam)3]^3+^ and [La(pcam)_3_X]^2+^ complexes acquired at the locations shown with dots in (c). The Au(111) surface state, SS, is also recorded in both spectra at −0.45 V. Notice that the LUMO is absent in the [La(pcam)_3_X]^2+^ complex. The upper spectrum is vertically shifted for clarity.

The observed ionic clusters are still mobile on the Au(111) surface even at 5 K when scanning with a lower bias (0.25 V). An example is shown in **Figure** **4**a,b, where a four‐unit cluster is moved during imaging producing an abrupt change (indicated with an arrow in Figure 4a). When the same area is imaged in a consecutive scan, the cluster has moved laterally, but it remains intact. Indeed, the clusters are mobile on the surface and the movements proceed with the entire cluster. In all experiments, we have not observed any separation of the clusters during motions. Moreover, attempts to separate the molecules by applying high voltage pulses exceeding ±3 V have not been successful, and the entire cluster moves away from the STM tip. This is unusual, as in other molecular assemblies the binding between molecules is generally weak, and an STM tip can easily extract individual molecules.^[^ ^24^ ^]^

Next, we determine the electronic structures of [La(pcam)_3_]^3+^ and [La(pcam)_3_X]^2+^ complexes by using d*I/*d*V* tunneling spectroscopy. Two‐ and four‐unit clusters are mobile during the spectroscopic measurement using biases exceeding 1 V, and only six‐unit clusters are found to be less mobile. Therefore, the d*I/*d*V* spectra and spectroscopic maps are acquired on a six‐unit cluster for a bias range between −2.0 and +1.5 V. An STM image of a six‐unit cluster together with a simultaneously acquired d*I/*d*V* map at 1 V bias is shown in Figures 4c,d respectively. Despite the STM image revealing both species, interestingly only [La(pcam)_3_]^3+^ provides the d*I/*d*V* contrast in the d*I/*d*V* map at 1 V (see a spectroscopic movie in Supporting Information). For comparison, point d*I/*d*V* spectra acquired over [La(pcam)_3_]^3+^ and [La(pcam)_3_X]^2+^ complexes are presented in Figure 4e. Here, the highest occupied molecular orbital (HOMO) of both [La(pcam)_3_]^3+^ and [La(pcam)_3_X]^2+^ complexes are located at ≈ −1.7 eV. However, the lowest occupied molecular orbital (LUMO) is observed only on [La(pcam)_3_]^3+^ at +1 eV in this energy range. Thus, the HOMO–LUMO energy gap of the [La(pcam)_3_]^3+^ is 2.7 eV. Since the LUMO state is absent in the [La(pcam)_3_X]^2+^ complex within this energy range, its d*I/*d*V* contrast is not observed for the complex in the spectroscopic map (Figure 4d).

**Figure 5:**
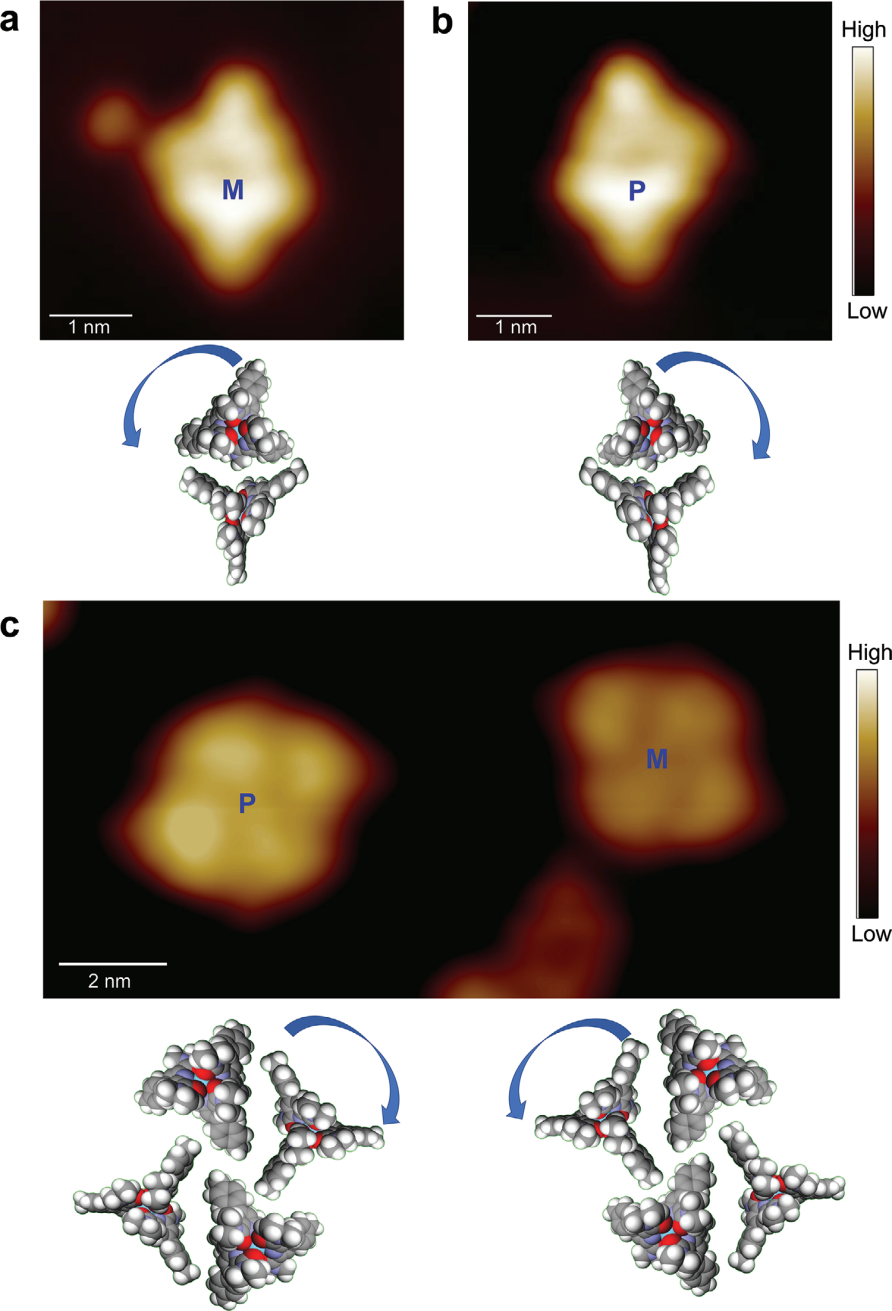
Cluster chirality. a,b) STM images of two‐unit cluster enantiomers and corresponding models. (Tunneling parameters: *V*_t_ = 0.5 V, *I*_t_ = 3.3 × 10^−11^ A for (a), *V*_t_ = 0.25 V, *I*_t_ = 2.0 × 10^−11^ A for (b)). c) An STM image showing a pair of four‐unit cluster enantiomers and corresponding models. (Tunneling parameters: *V*_t_ = 1.0 V, *I*_t_ = 9.5 × 10^−12^ A). “M” and “P” labeling is arbitrary, as the chirality of each unit in the cluster cannot be assessed by STM.

STM images reveal that the clusters are chiral.^[^ ^25^ ^]^ **Figure** **5**a,b presents a pair of enantiomers of two‐unit clusters. Here, two arrangements for the pairing of [La(pcam)_3_X]^2+^ to [La(pcam)_3_]^3+^ lead to different enantiomers. Propagation of chirality results in the formation of enantiomers for the basic unit complex pairs, as shown in Figure 5a,b. Similarly, the four‐unit cluster is also chiral and exists as a racemic mixture of enantiomers as shown in Figure 5c. In principle, the six‐unit cluster should also be a mixture of enantiomers, but their limited number on the surface prevents a full characterization. Regardless of chirality, the observed rare‐earth ionic clusters are mobile on the Au(111) surface at 5 K, and both chiral clusters are strictly stable and do not interconvert into their enantiomeric forms.

## 3 Conclusion

We report the formation of charged rare‐earth ionic clusters on a metal surface. Here, the La ion coordinated in complex [La(pcam)_3_]^3+^ is well protected from the environment by the ligand. Even after absorption on a Au(111) surface, only a negligible amount of charge transfer occurs between the substrate and the complex, and as a result, the La ion remains closed to its +3 charged state almost as in the gas phase. Similarly, the triflate anion maintains its negative charge upon the formation of complex [La(pcam)_3_X]^2+^ on Au(111). The withholding of their charged states on Au(111) surface leads to the formation of ionic clusters with a basic unit composed of a pair of [La(pcam)_3_]^3+^ and [La(pcam)_3_X]^2+^ complexes, and strongly screened by the electrons in the metal. Unlike their non‐ionic counterparts on surfaces, the electric‐field–induced manipulation with the STM tip further highlights a strong interaction between these ionic clusters; density functional theory also supports this assessment. Furthermore, using tunneling spectroscopy and spectroscopic mapping, we have investigated their electronic states inside a cluster with sub‐molecular scale spatial resolution. Moreover, their high mobility on the surface, even at the low substrate temperature of 5 K, indicates they might be considered precursors to form an ionic liquid state. Since ionic liquids are used in many applications spanning semiconductors,^[^ ^26^ ^]^ batteries,^[^ ^27^ ^]^ perovskite solar cells,^[^ ^28^ ^]^ and medicine,^[^ ^19^ ^]^ the finding reported here may have an impact on potential applications.

## 4 Experimental Section

### STM Experiments

Au(111) and Cu(111) single crystal surfaces were cleaned by repeated cycles of sputtering with Ar ions and annealing under an ultrahigh vacuum (UHV) environment. The cleanliness of the samples was checked by means of STM imaging. Next, sub‐monolayer coverages of the La(pcam)_3_ triflate salt were deposited onto the atomically clean samples held at room temperature via thermal deposition under UHV. The samples were transferred to the STM scanner under UHV directly attached to the preparation chamber via a gate valve. STM imaging of the individual species on Cu(111) was performed at 5 K substrate temperature. The salt deposited onto Cu(111) surface was used only for the structural analysis of the molecular species. On Au(111) surface, the STM imaging was performed at ≈120 and 5 K substrate temperatures. Tunneling spectroscopy, as well as electric field manipulation, were performed on the sample deposited on Au(111) surface at 5 K. Tunneling spectroscopy and spectroscopic maps were measured by using a lock‐in amplifier. A lock‐in voltage modulation between 10 and 20 mV with a frequency range of 800 to 1000 Hz was added to the STM tip for the spectroscopic measurements.

### DFT Calculations

DFT calculations to determine the structure of complexes in the gas phase as well as after adsorption on Au(111) surface were performed by using the Vienna ab initio simulation package,^[^ ^29^, ^30^, ^31^, ^32^ ^]^ and the core electrons were described by the projected augmented wave method.^[^ ^33^ ^]^ Exchange correlation was treated in the generalized gradient approximation, as implemented by Perdew et al.^[^ ^34^ ^]^ The plane wave basis was expanded to a kinetic energy cut‐off of 400 eV for the complex pair calculation while 600 eV was used for the rest. The energy cut‐off used here was enough to describe the electronic structure since La f‐bands were fully empty. The Brillouin zone was sampled using Γ points only. Because of the relative importance of non‐bonding complex surface interactions, the van der Waals D3 dispersion term was applied.^[^ ^35^ ^]^ The GGA+*U* scheme within the rotationally invariant formalism was used together with the fully localized limit double‐counting formula, using *U*_d_(Au) = 3 and *U*_d_(La) = 5. It was known that La d bands can be too low within GGA, and thus, nonzero *U*_d_(La) was needed to describe the electronic structure properly near the Fermi level.^[^ ^36^ ^]^ For the slab calculation of molecules on 3 Au atomic layers (514 atoms for the single complex calculations, and 720 atoms for the RE cluster calculations), dipole correction was used to avoid an artificial dipole field in the supercell.^[^ ^37^ ^]^ Geometrical relaxation was terminated when the change of the total energy was smaller than 1 × 10^−4^ eV between two ionic steps.

## Conflict of Interest

The authors declare no conflict of interest.

## Author Contributions

S.W.H. designed the experiments; E.M. designed the rare‐earth complexes; D.T., S.S., S.W., T.M.A., S.P., and K.Z.L. performed STM experiments; D.T., S.S., S.W., T.M.A., S.P., K.Z.L., and S.W.H. analyzed the experimental data; X.C. synthesized the complexes; A.T.L., V.S., D.N., L.A.C, D.F., S.E.U., and A.T.N. performed the calculations, and S.W.H. and E.M. wrote the manuscript. All the authors discussed the results and commented on the manuscript.

## Supporting information

Supporting Information

Supplemental Movie 1

## Data Availability

The data that support the findings of this study are available from the corresponding author upon reasonable request.
