# Supplementary material for: 2D Ionic Liquid‐Like State of Charged Rare‐Earth Clusters on a Metal Surface
Source: Adv Sci (Weinh). 2024 Jan 24;11(13):2308813. doi: 10.1002/advs.202308813 (PMC10987101; doi:10.1002/advs.202308813)
Supplement: Supplementary file 1 — Supporting Information [file ADVS-11-2308813-s001.pdf]

ADVANCED SCIENCE
Open Access

Supporting Information

for *Adv. Sci.*, DOI 10.1002/advs.202308813

2D Ionic Liquid-Like State of Charged Rare-Earth Clusters on a Metal Surface

*Daniel Trainer, Alex Taekyung Lee, Sanjoy Sarkar, Vijay Singh, Xinyue Cheng, Naveen K. Dandu, Kyaw Zin Latt, Shaoze Wang, Tolulope Michael Ajayi, Sineth Premarathna, David Facemyer, Larry A. Curtiss, Sergio E. Ulloa, Anh T. Ngo, Eric Masson and Saw Wai Hla**

Supplementary Information

2-D Ionic Liquid-Like State of Charged Rare-Earth Clusters on a Metal Surface

Daniel Trainer, Alex Taekyung Lee, Sanjoy Sarkar, Vijay Singh, Xinyue Cheng, Naveen K. Dandu, Kyaw Zin Latt, Shaoze Wang, Tolulope Michael Ajayi, Sineth Premarathna, David Facemyer, Larry A. Curtiss, Sergio E. Ulloa, Anh T. Ngo, Eric Masson, & Saw Wai Hla

Contents

S.1. Molecular Dimension 2
S.2. Two-Unit Cluster Mobility 3
S.3. Four-Unit Cluster Mobility 4
S.4. Calculated Adsorption Geometries and Valence Charges 5
References 6

The submitted manuscript has been created by UChicago Argonne, LLC, Operator of Argonne National Laboratory ("Argonne"). Argonne, a U.S. Department of Energy Office of Science laboratory, is operated under Contract No. DE-AC02-06CH11357. The U.S. Government retains for itself, and others acting on its behalf, a paid-up nonexclusive, irrevocable worldwide license in said article to reproduce, prepare derivative works, distribute copies to the public, and perform publicly and display publicly, by or on behalf of the Government.

S1. Molecular Dimensions

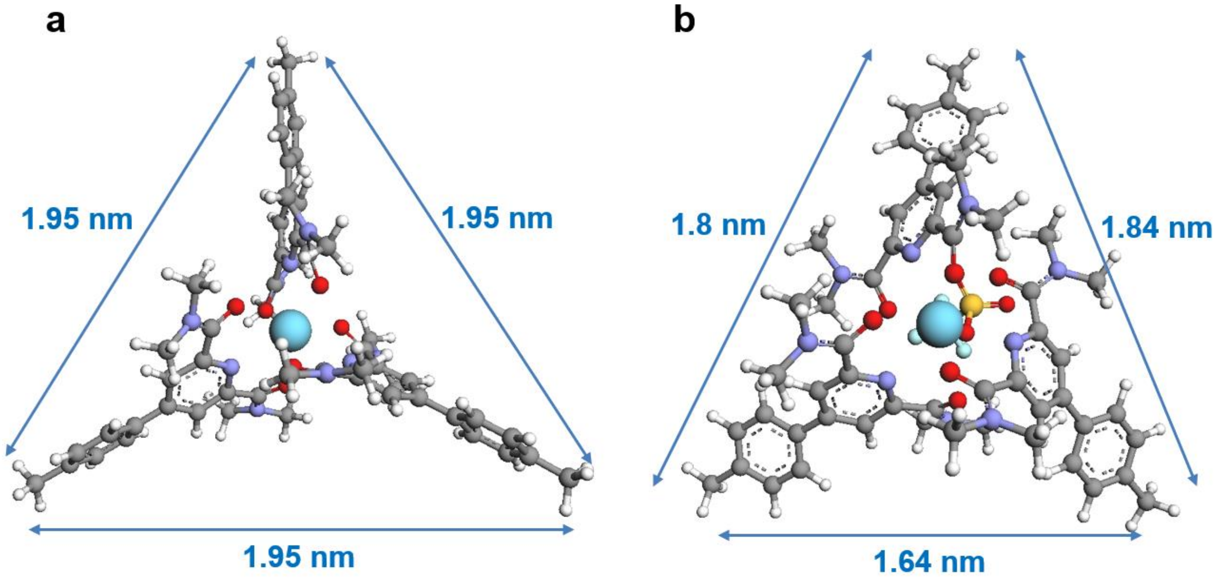

Figure S1. *Molecular Dimensions.* ***(a)*** *[La(pcam)₃]*$^{3+}$*, and* ***(b)*** *[La(pcam)₃X]*$^{2+}$ *complex.*

$[La(pcam)_3]^{3+}$has a uniform triangular shape with a distorted D_{3h} geometry and an average side length of 1.95 nm (Fig. S1a). The $[La(pcam)_3X]^{2+}$ complex has a distorted triangular geometry and one of its sides is reduced to 1.64 nm while the other side lengths are 1.8 nm and 1.84 nm, respectively.

S2. Two-Unit Cluster Mobility

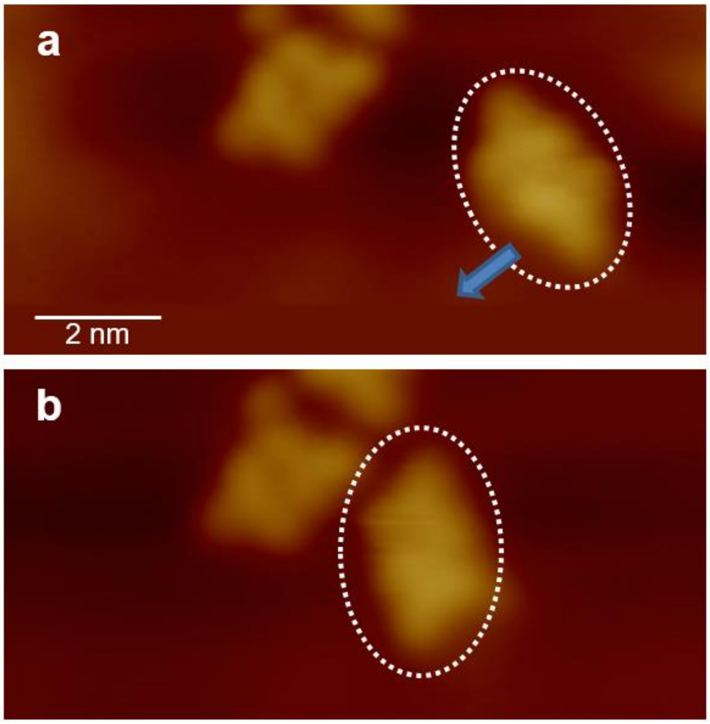

Figure S2. *A sequence of STM images reveals two-unit cluster mobility. [Tunneling parameters:* $V_t = 0.25$ *V,* $I_t = 2.0 \times 10^{-11}$*A, 5K].*

S3. Four-Unit Cluster Mobility

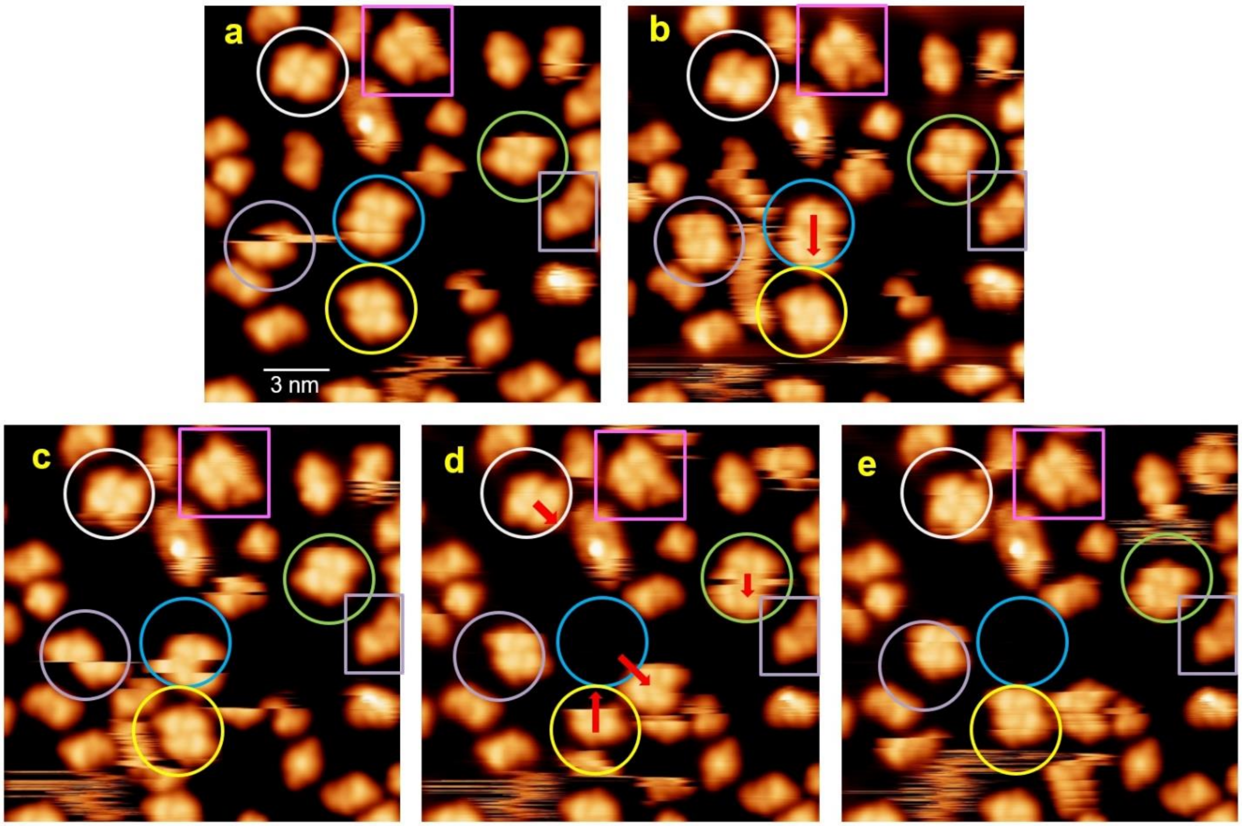

Figure S3. *A sequence of STM images reveals four-unit cluster mobility.*
[Tunneling parameters: V_t = 1.0V, I_t = 3.8 x 10^{-11}A, 5K].

Figure S3 shows a sequence of STM images in which the locations of four-unit clusters are marked with colored ovals. The STM images recorded the mobilities of all four-unit clusters however, the clusters remain intact. In addition, a five-unit aggregate (a 4-unit cluster +1, pink square oval), and a three-unit aggregate (2-unit cluster +1, violet rectangular oval) are shown. These aggregate are not mobile and remain stationary.

S4. Calculated Adsorption Geometries and Valence Charges

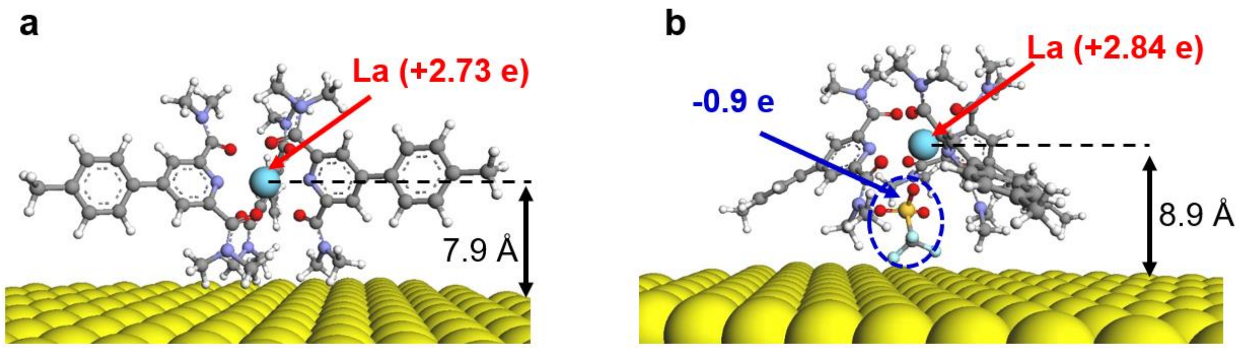

***Figure S4. a**, Sideviews of La(pcam)$_3$ and **b**, La(pcam)$_3$ X on Au(111).*

Figure S4 shows the sideviews of the calculated adsorption geometries of $[La(pcam)_3]^{3+}$ and $[La(pcam)_3X]^{2+}$ (where X = $CF_3SO_3^-$). Geometrically relaxed density functional theory calculations were performed by using VASP code and positioning the molecule and the complex on a 3-atomic layer Au(111) slab representing the surface. The calculations reveal that the La ion in the La(pcam)$_3$ molecule is located 7.9 Å above the Au(111) surface (Fig. S4a). For the $[La(pcam)_3X]^{2+}$ complex, the incorporation of the triflate ion (indicated with the dashed oval in Fig. S4b) causes the molecule to bend upward and the La ion is located 8.9 Å above the surface. Both La ion distances from the surface are too far to interact directly with the substrate.

Next, the valence charges of the $[La(pcam)_3]^{3+}$ and $[La(pcam)_3X]^{2+}$ complex in the gas phase as well as the ones adsorbed on Au(111) surface are calculated using the method developed by

Henkelman group[1,2,3] by partitioning a charge density grid into Bader volumes. The calculated results are shown in table 1 of the main text.

References

[1] W. Tang, E. Sanville, E., G. Henkelman, *J. Phys.: Condens. Matter* **2009**, *21*, 084204.

[2] E. Sanville, S. D. Kenny, R. Smith, G. Henkelman, *J. Comp. Chem.* **2007**, *28*, 899.

[3] G. Henkelman, A. Arnaldsson, H. A. Jónsson, *Comput. Mater. Sci.* **2006**, *36*, 354.
